# Whole exome sequencing and bioinformatics reveal PMAIP1 and PDGFRL as immune-related gene markers in follicular thyroid carcinoma

**DOI:** 10.3389/fgene.2025.1509245

**Published:** 2025-05-22

**Authors:** Haobo Wang, Fangjian Shang, Xia Jiang, Zhongxin Li, Dongyun Li, Chuanmin Zhou, Bo Pang, Longfei Kang, Bo Liu, Zengren Zhao

**Affiliations:** ^1^ Department of General Surgery, The First Hospital of Hebei Medical University, Shijiazhuang, China; ^2^ Gastrointestinal Disease Diagnosis and Treatment Center, Hebei Key Laboratory of Colorectal Cancer Precision Diagnosis and Treatment, The First Hospital of Hebei Medical University, Shijiazhuang, China

**Keywords:** follicular thyroid carcinoma, follicular thyroid adenoma, PMAIP1; PDGFRL, whole exome sequencing, bioinformatics

## Abstract

**Introduction:**

The primary clinical challenge associated with follicular thyroid carcinoma (FTC) lies in accurately diagnosing the condition, particularly in distinguishing it with follicular thyroid adenoma (FTA) due to their overlapping cytomorphological features and sonographic characteristics.

**Methods:**

Whole exome sequencing (WES) techniques and Gene Expression Omnibus (GEO) database were utilized to analyze genomic difference between FTC and FTA, with a specific focus on immune-related genes. The hub genes were subjected to enrichment analysis, immune infiltration analysis, protein-protein interaction (PPI) analysis, and receiver operating characteristic (ROC) curve analysis. Then utilized quantitative real-time polymerase chain reaction (qRT-PCR) and immunohistochemistry (IHC) to validate the expression levels of PMAIP1 and PDGFRL at the cellular and tissue levels.

**Results:**

The findings of WES and bioinformatics analysis indicated that PMAIP1 and PDGFRL were potential mutated immune-related genes in FTC, in comparison to FTA, the expression of PMAIP1 is up-regulated in FTC while PDGFRL is down-regulated, demonstrating promising diagnostic efficacy. Enrichment analysis and immune infiltration analysis suggested that PMAIP1 and PDGFRL may serve as potential therapeutic targets for FTC. The results of the validation at both cellular and tissue levels indicated an up-regulation of PMAIP1 and a down-regulation of PDGFRL in FTC, consistent with the results from bioinformatics analysis.

**Discussion:**

In conclusion, it is the first research to revealed PMAIP1 and PDGFRL as potential novel immunodiagnostic markers for FTC, shedding light on their potential biological significance in this context, and offering potential valuable clinical applications.

## Introduction

Thyroid cancer (TC) stands as the most prevalent type of endocrine malignancy. Its incidence has notably surged over the past 3 decades, making it the most frequently diagnosed cancer among individuals aged 15–29 years, and the second most prevalent among those aged 30–39 years (Kitahara and Sosa, 2020; Miller et al., 2020). Furthermore, TC holds the 11th position in both incidence rate and mortality among all cancer types, underscoring the need for heightened awareness and attention to this disease (Sung et al., 2021). Follicular thyroid carcinoma (FTC) constitutes a distinct subset of thyroid cancers, with an incidence rate that, while lower than that of papillary thyroid carcinoma (PTC), still accounts for a significant 10%–15% of cases. Notably, FTC was characterized by a more aggressive clinical behavior compared to its papillary counterpart (Daniels, 2018). It is noteworthy that FTC often presents with metastases in distant organs, such as the lungs or bone, in 10%–15% of patients (Castellana et al., 2020). Consequently, despite its lower incidence, FTC is associated with a more adverse prognosis.

Originating from follicular cells, FTC was characterized by a propensity for capsular and vascular invasion. The clinical complexity of diagnosing FTC lies in its precise identification, particularly in distinguishing it from follicular thyroid adenoma (FTA) and assessing for histological evidence of capsular or vascular invasion—a formidable task even for seasoned pathologists (Heikkila et al., 2013). FTA, a benign adenoma originating from follicular cells, contrasts significantly with FTC due to the absence of capsular invasion, vascular invasion, extrathyroidal tumor extension, or metastases. Despite these distinctions, the similar cytomorphological features of FTC and FTA make differentiation challenging (Asa and Mete, 2013; Xu and Ghossein, 2023). Additionally, not all FTC clinically exhibit vascular and capsular invasion, further complicating the distinction between the two entities. In cases where an accurate diagnosis cannot be achieved through intraoperative frozen-section analysis, a pathologist may lean towards a benign diagnosis due to the high prevalence of benign nodules and the influence of experience (Levi et al., 2013). While FTC can be definitively diagnosed through histological examination following partial or complete thyroidectomy, the importance of preoperative diagnosis should not be understated in guiding surgical decision-making, including the selection of appropriate surgical techniques and the determination of the extent of resection (Zheng et al., 2022). Despite the evolution in the histological categorization of FTC over the past 50 years, achieving a precise diagnosis remains a complex endeavor (Cipriani et al., 2015).

Therefore, it is imperative to accurately diagnose FTC and FTA prior to surgery. Notably, Fine-needle aspiration cytology (FNAC) and ultrasonography (US) are frequently utilized clinical detection methods for auxiliary diagnosis. However, they are not capable of providing a precise diagnosis of FTC. The limited accuracy of FNAC is attributed to its inability to diagnose based solely on cell morphology (Soares et al., 2020). Additionally, the sonographic characteristics of FTA and FTC exhibited significant similarities, with substantial overlap in ultrasonographic features, typically presenting as well-defined, solid, homogeneous, hypoechoic, or isoechoic nodules with a peripheral halo (Soares et al., 2022). Consequently, the efficacy of this method for distinguishing between FTC and FTA may be limited.

Hence, there is a pressing need to explore novel approaches for the diagnosis of FTC. Recent research has highlighted the potential utility of genetic alterations in TC as promising targets for diagnostic and therapeutic interventions (Cabanillas, McFadden, and Durante, 2016). Furthermore, the presence of aggressive follicular cell-derived thyroid cancer, either at the time of initial diagnosis or during subsequent monitoring, has been linked to the suppression of T cell subsets, including CD4^+^ T cells, gamma-delta T cells, and NK T-like cells, as well as the upregulation of myeloid-derived suppressor cells and alterations in memory T cells (Kotwal et al., 2024). These immune-related markers may serve as potential targets for the diagnosis and treatment of FTC.

**FIGURE 1:**
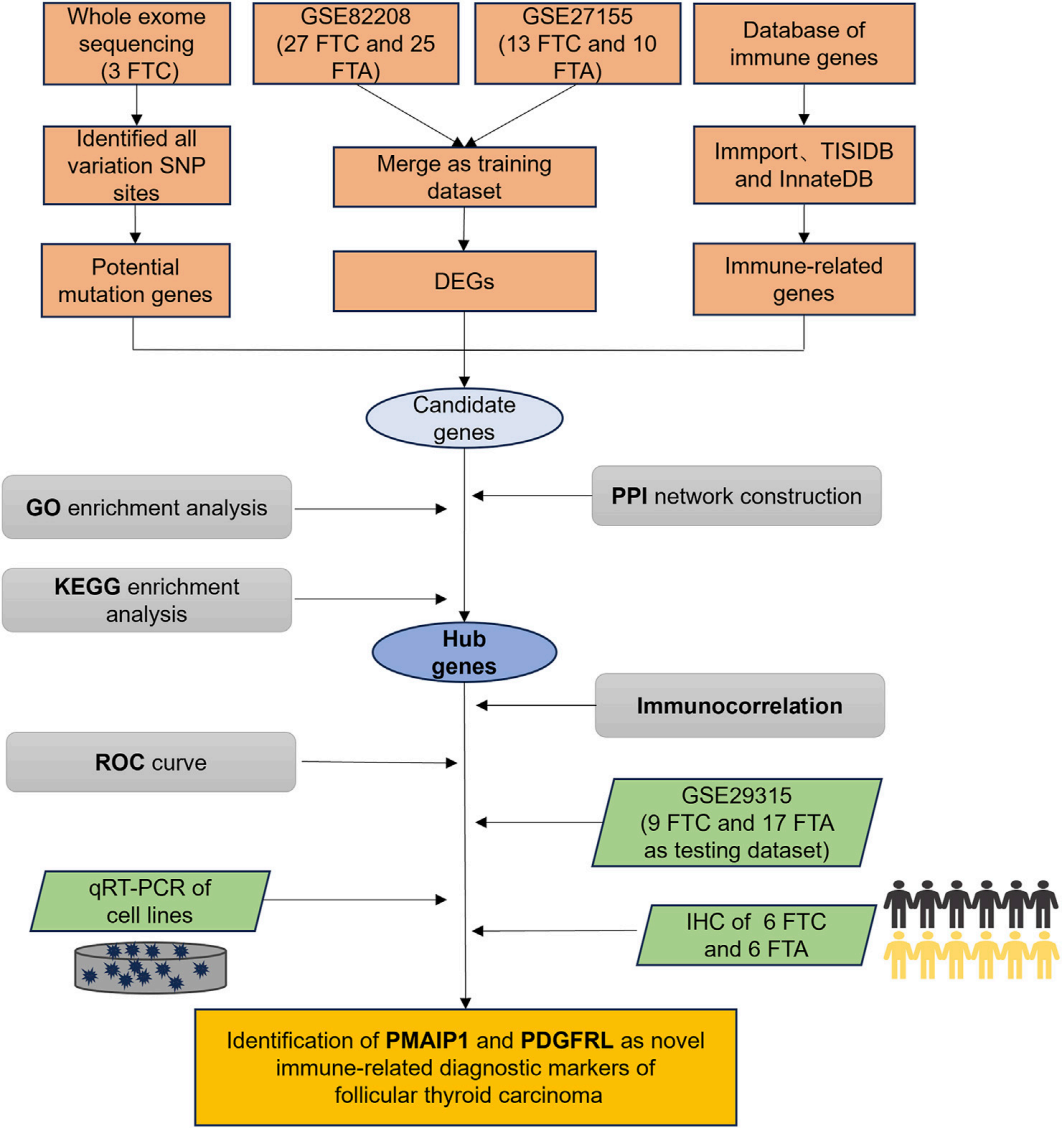
The flow chart of our study.

This study utilized whole exome sequencing (WES) to detect potential single nucleotide polymorphism (SNP) mutation sites in FTC and provide annotations. Concurrently, bioinformatics tools were employed to investigate the differentially expressed genes (DEGs) between FTC and FTA, in conjunction with immune gene sets for analysis. Subsequently, the potential functions, immune relevance, and diagnostic efficacy of these genes were assessed. Validation experiments were then conducted at the cellular and tissue levels to confirm the expression levels, revealing phorbol-12-myristate-13-acetate-induced protein 1 (PMAIP1) and platelet derived growth factor receptor like (PDGFRL) as potential immune-related diagnostic markers for FTC (Figure 1).

## Materials and methods

### Ethics statement

All patients received appropriate and professional medical treatment. This study was ethically approved by the Ethics Committee of the First Hospital of Hebei Medical University (NO: 20230203), and all patients provided informed consent for the collection of tissue samples and analysis of clinical information.

### Clinical samples

3 patients with FTC who underwent surgical treatment at our hospital were selected for WES analysis. Additionally, formalin-fixed, paraffin-embedded (FFPE) tissue sections from 6 FTC patients and 6 FTA patients were utilized for further validation. Pathological diagnosis of all tissues was conducted by a minimum of two senior pathologists.

### WES and SNP variant sites detection

Following the extraction of DNA from FFPE of FTC, monitoring of DNA degradation and contamination was conducted on 1% agarose gels, while DNA concentration was assessed utilizing the Qubit^®^ DNA Assay Kit in the Qubit^®^ 3.0 Flurometer (Invitrogen, United States). DNA samples meeting the criteria of a DNA concentration of ≥20 ng/μL and a total amount exceeding 0.4 μg were selected for library construction. The enrichment of exome sequences was effectively achieved from 0.4 μg of genomic DNA utilizing the Agilent liquid capture system (Agilent SureSelect Human All Exon V6). Initially, high-quality genomic DNA was randomly fragmented to an average size of 180-280bp followed by end repair and phosphorylation of DNA fragments. Finally, the index-coded samples were clustered using an Illumina PE Cluster Kit on a cBot Cluster Generation System. Subsequently, the DNA libraries were sequenced on an Illumina platform, producing 150 bp paired-end reads.

The effective sequencing data were aligned with the human reference genome (GRCh37) using the Burrows Wheeler Aligner software to detect the variant information in the samples. Then, the alignment results were sorted using Sambamba. Variant calling and identification of SNPs was conducted using SAMtools mpileup. and the ANNOVAR software was used to annotate the SNPs. The annotation information included data from databases such as dbSNP, the 1000 Genomes Project, and other existing databases. The annotation content covered the location information, type, conservation prediction, etc. of the variants. Based on the alignment results, we used SAMtools to identify SNP sites, and adopted the internationally accepted filtering criteria (such as sequencing depth, site quality information, etc.) to filter these SNP sites. Remove the mutations with a frequency higher than 1% in at least one of the four frequency databases, namely, the 1,000 Genomes data, the ESP6500 database, and the gnomAD data. The aim is to remove the diversity sites among individuals and obtain the rare mutations that may be pathogenic.

### Acquisition of microarray data and immune-related genes

Specifically, cancer chip expression data sets GSE82208, GSE27155, and GSE29315 were downloaded from the Gene Expression Omnibus (GEO; https://www.ncbi.nlm.nih.gov/geo/) database. GSE82208 contained 27 FTC tissues and 25 FTA tissues, GSE27155 contained 13 FTC tissues and 10 FTA tissues, GSE29315 contained 9 FTC tissues and 17 FTA tissues. To reduce the confounding effects caused by data from different batches (GSE82208 and GSE27155), the ComBat algorithm was adopted, and the data was batch-corrected using the sva package in the R (Yu et al., 2024). Through the above operations, the two groups of GEO datasets can be integrated, and the subsequent analysis can be carried out. Additionally, 2,704 immune-related gene sets were retrieved from databases such as Immport, TISIDB, and InnateDB. The data was read and analyzed by using the Limma R package and ggplot2 package of the R.

### Enrichment analysis

The genes were subjected to Kyoto Encyclopedia of Genes and Genomes (KEGG) and Gene Ontology (GO) analyses using the ClusterProfiler R package to assess statistical enrichment in gene functions and biological pathways. The GO annotation included cellular component (CC), biological process (BP), and molecular function (MF).

### Protein-protein interaction (PPI) analysis

Candidate genes were analyzed for PPI using the Search Tool for the Retrieval of Interacting Genes (STRING) database (https://cn.string-db.org).

### Immune cell correlation analysis

RNAseq data and corresponding clinical information from 106 cases of FTC were obtained from the TCGA database (https://www.cancer.gov/ccg/research/genome-sequencing/tcga). The immune cell correlation of hub genes was analyzed utilizing the MCP counter algorithm of the R immunedeconv package, and a multi-gene correlation map was generated using the R pheatmap package. Spearman’s correlation analysis was employed to assess the relationship between quantitative variables that did not follow a normal distribution. *P* < 0.05 was deemed statistically significant.

### Diagnostic performance analysis

Take the GSE82208 and GSE27155 datasets that have been processed for batch effect removal as the data sets, and use the pROC software package to evaluate the diagnostic performance of the hub genes, with the area under the curve (AUC) defined as the area enclosed by the coordinate axis under the receiver operating characteristic (ROC) curve, where AUC values range from 0.5 to 1. The higher the AUC value approaches 1.0, the greater the reliability of the detection method.

### Cell culture

The human FTC orthotopic cell lines FTC133 (1 × 10^6^) and the normal thyroid cell line NTHY-ORI3-1 (1 × 10^6^) were procured from Procell Life Science&Technology Co, Ltd. (Cat NO.: CL-0644; CL-0817). FTC lung metastasis cell line FTC238 (1 × 10^6^) were procured from YUCHI Biology Co, Ltd. (Cat NO.: SC-1458). FTC238 was maintained in DMEM F-12 medium (Thermo, Gibco, United Kingdom), while FTC133 and NTHY-ORI3-1 were cultured in RPMI-1640 medium (Thermo, Gibco, United Kingdom), all supplemented with 10% fetal bovine serum (Thermo, Gibco, United Kingdom) and 1% penicillin-streptomycin (Thermo, Gibco, United Kingdom). All cells were incubated at 37°C with 95% humidity and 5% CO_2_.

### Quantitative real-time polymerase chain reaction (qRT-PCR)

Total RNA extraction was performed using RNA EASY reagent (R701-01, Vazyme, China), followed by reverse transcription using the Prime Script RT Reagent Kit (RR047A, TaKaRa, Japan). The chamQ universal SYBR qPCR master mix (Q711, Vazyme, China), cDNA, and primer were used for qRT-PCR system (LightCycler 480Ⅱ, United States). PCR amplification reactions were performed in triplicates for each cDNA sample. β-actin was used as an internal reference. All gene-specific primers were designed using NCBI (https://www.ncbi.nlm.nih.gov/) and synthesized by sangon biotech Co, Ltd. (shanghai) (Table 1). The relative expression was estimated by 2^−ΔΔCT^ method.

**TABLE 1:** Primer sequences used in this study.

Gene name	Primer sequences	Length (bp)
TNFRSF11B	F: GGA​ACC​CCA​GAG​CGA​AAT​ACA	212
	R: TCC​TCA​CAC​AGG​GTA​ACA​TCT​ATT​C	
CXCL12	F: CTG​TGC​CCT​TCA​GAT​TGT​AGC​C	150
	R: TGT​AAG​GGT​TCC​TCA​GGC​GT	
EEF1A2	F: GTC​AAG​GAA​GTC​AGC​GCC​TA	126
	R: CTT​GAA​CCA​CGG​CAT​GTT​GG	
PDGFRL	F: ACT​CAG​CCA​ATT​CAG​CAC​CA	201
	R: CAT​TCT​GCT​TGA​CGC​TGA​GG	
PMAIP1	F: AGG​AAC​AAG​TGC​AAG​TAG​CTG	153
	R: GGA​GTC​CCC​TCA​TGC​AAG​TT	
β-actin	F: ACT​TAG​TTG​CGT​TAC​ACC​CTT	155
	R: GTCACCTTCACCGTTCCA	

### Immunohistochemical (IHC) staining and quantitative analysis

The rabbit polyclonal antibody against PMAIP1 (A9801) was from ABclonal Technology Co, Ltd. (Wuhan, China). The mouse monoclonal antibody against PDGFRL (sc-393355) was from Santa Cruz Biotechnology Co, Ltd. (Shanghai, China). All antibodies were diluted at a ratio of 1:50. Following dewaxing and rehydration, tissue sections were immersed in 0.01M citrate buffer and heated in a microwave to restore antigenicity. After incubating at room temperature for 10 min, the sections were treated with PBS and 1% periodate. Subsequently, the sections were exposed to the primary antibody overnight at 4°C before undergoing incubation with the secondary antibody for 30 min the following day. The sections underwent a staining process involving DAB, PBS washing, hematoxylin counterstaining, distilled water washing, and PBS rebluing. Dehydration was achieved using gradient alcohol, with 5-min intervals per stage, followed by a 10-min xylene treatment. Finally, neutral gum was applied to seal the sections for observation under a microscope. Subsequently, three images of each tissue slice were randomly captured under a microscope, and the staining intensity of the images was analyzed using Aipathwell software (Wuhan servicebio technology Co, Ltd.). Staining intensity of sections was assessed by H-score. H-score was calculated by using the formula “H-SCORE = ∑ (pi × i) = (percentage of weak intensity × 1) + (percentage of modal intensity × 2) + (percentage of strong intensity × 3)”.

### Statistical analysis

Data analysis was performed using SPSS 22.0 software (SPSS Inc., Chicago, United States), with bioinformatics analysis conducted using R (version 4.3.0). If the original data followed a normal distribution, statistical descriptions were presented as mean ± standard deviation (±S), and t-tests were used for statistical analysis. If the original data deviated from a normal distribution, statistical description utilized the median and interquartile range, while statistical analysis employed the rank sum test. Statistical significance was determined at a *P* < 0.05.

## Results

### Clinicopathologic characteristics of patients

The clinicopathologic characteristics of three patients with FTC who participated in the WES study was presented (Table 2). The cohort consisted of two females and one male, with tumors located in various regions of the thyroid gland, all exhibiting intravascular cancer thrombus. Notably, not all tumors demonstrated capsular invasion and necrosis, and tumor size was found to be positively correlated with patient age.

**TABLE 2:** Clinicopathologic characteristics of patients.

Patient	Gender	Age	Location	Tumor size (mm)	Tumor volume (mm^3^)	Envelope violation	Necrosis	Intravasc-ular cancer thrombus
1	Male	67	Left lobe and isthmus	8 × 8 × 8	512	√	√	√
2	Female	50	Right lobe	4.8 × 4.5 × 3	64.8	√	×	√
3	Male	41	Right lobe and isthmus	5 × 3 × 2.5	37.5	×	√	√

### Sequencing data and mutation SNP sites

Quality statistics of the sequencing data are detailed (Table 3). The WES analysis resulted in an average of 27.64 GB of clean data, with 93.28% of reads having a quality score of Q30 and an average mapping rate of 99.42%. Additionally, an average sequencing depth of 319.16X and a target region coverage of 99.6% were achieved. These results indicate that the sequencing depth and coverage of this study are superior to conventional sequencing methods, allowing for the detection of more mutation sites and the acquisition of more comprehensive information. Furthermore, the data quality meets the necessary standards for analysis. The SNP data obtained from the initial analysis underwent filtering based on mutation sites, leading to the annotation of variant sites. By incorporating SNP annotation data that has been filtered and screened through the aforementioned processes, a total of 7,230 genes with potential significance were identified.

**TABLE 3:** The quality of sequencing data.

Sample	Total	Raw data(G)	Q30 (%)	Properly mapped (%)	Average sequencing depth	Coverage of target region (%)
1	178,664,266	27.08	93.09	99.44	311.84	99.5
2	192,717,782	29.25	93.34	99.31	343.81	99.6
3	175,054,238	26.59	93.42	99.53	301.84	99.8
Mean	182,145,429	27.64	93.28	99.42	319.16	99.6

### Identification and analysis of DEGs

**FIGURE 2:**
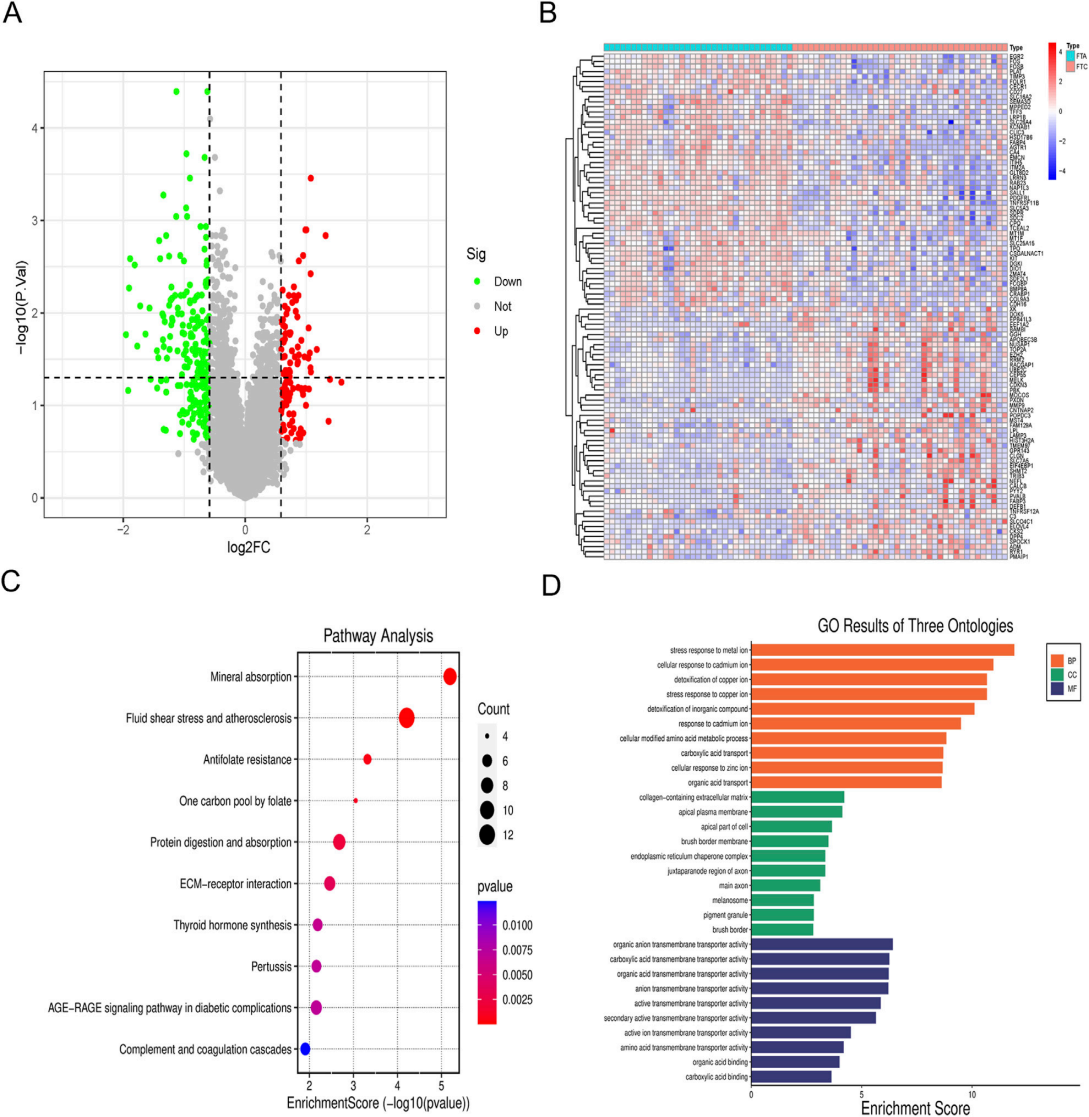
Analysis of DEGs. **(A)** Volcano map of DEGs; **(B)** Cluster heat map of the top 100 DEGs; **(C)** KEGG pathway enrichment analysis of DEGs; **(D)** GO enrichment analysis of DEGs.

The chip expression data GSE82208 and GSE27155 underwent preprocessing and normalization procedures by R. DEGs were defined by simultaneously meeting the criteria of fold change = 1.5 and p-value<0.05. The identification and analysis of DEGs yielded a total of 332 genes, comprising 111 upregulated genes and 221 downregulated genes (Figure 2A). The chip expression data was sorted based on P values, and a cluster heat map of the top 100 DEGs was presented (Figure 2B). KEGG enrichment analysis revealed significant associations with fluid shear stress and atherosclerosis, mineral absorption pathways (Figure 2C). The DEGs were found to be enriched in stress response to metal ion (ontology: BP), collagen-containing extracellular matrix (ontology: CC), and organic anion transmembrane transporter activity (ontology: MF) (Figure 2D).

### Identification and analysis of hub genes

**FIGURE 3:**
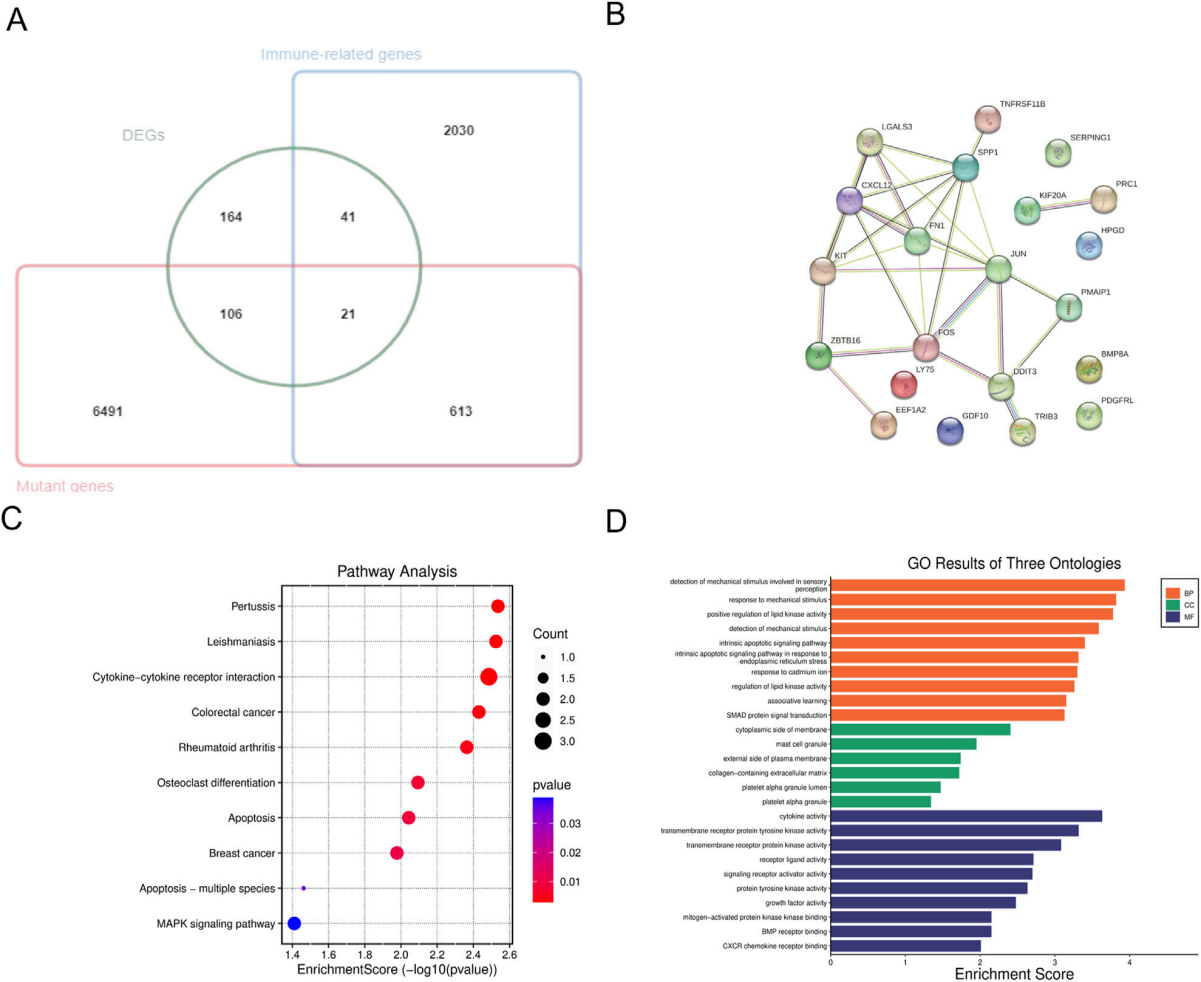
Analysis of hub genes. **(A)** The venn diagram of the DEGs, mutant genes and immune-related genes; **(B)** PPI analysis of candidate genes; **(C)** KEGG pathway enrichment analysis of hub genes; **(D)** GO enrichment analysis of hub genes.

A Venn diagram was utilized to compare mutated genes, DEGs, and immune-related genes, leading to the identification of 21 candidate genes associated with FTC and FTA (Figure 3A; Table 4). PPI analysis of these 21 genes were identified (Figure 3B). Furthermore, the top 10 genes were selected based on their |LogFC|, which were TNFRSF11B、KIT、FOS、BMP8A、PDGFRL、TRIB3、EEF1A2、PMAIP1、CXCL12 and SERPING1.Enrichment analysis revealed that these genes are primarily associated with cytokine-cytokine receptor interaction and MAPK signaling pathway (Figure 3C). Additionally, they were found to be enriched in detection of mechanical stimulus involved in sensory perception (ontology: BP), cytoplasmic side of membrane (ontology: CC), and cytokine activity (ontology: MF) (Figure 3D).

**TABLE 4:** Information of candidate genes.

	Gene name	|logFC|	logFC	t	*P* value
1	TNFRSF11B	1.886916105	−1.886916105	−4.772557911	0.000008852
2	KIT	1.768736408	−1.768736408	−3.628010825	0.000520467
3	FOS	1.56925219	−1.56925219	−4.158580349	0.000084740
4	BMP8A	1.246762878	−1.246762878	−4.202051224	0.000072595
5	PDGFRL	1.192155397	−1.192155397	−3.978037336	0.000159608
6	TRIB3	1.172688976	1.172688976	3.579734372	0.000609581
7	EEF1A2	1.069526898	1.069526898	3.302794039	0.001472376
8	PMAIP1	1.035114095	1.035114095	3.428347502	0.000992427
9	CXCL12	1.005115604	−1.005115604	−3.491107111	0.000812109
10	SERPING1	0.878347248	−0.878347248	−3.038419874	0.003278442
11	LY75	0.8639	−0.863899655	−2.446458879	0.016785577
12	JUN	0.821700771	−0.821700771	−3.871910062	0.000229894
13	KIF20A	0.735546597	0.735546597	3.366919561	0.001205051
14	HPGD	0.722749541	0.722749541	2.842711019	0.005768558
15	PRC1	0.67434	0.674340423	2.87985265	0.005191596
16	FN1	0.658180625	0.658180625	2.009715616	0.048082674
17	DDIT3	0.622324135	0.622324135	2.948816848	0.004259076
18	ZBTB16	0.618526202	−0.618526202	−2.477083896	0.015513662
19	GDF10	0.61661265	−0.61661265	−2.071860141	0.04173993
20	SPP1	0.597441017	0.597441017	2.192126331	0.031494108
21	LGALS3	0.59385185	0.59385185	2.603394475	0.011133859

**FIGURE 4:**
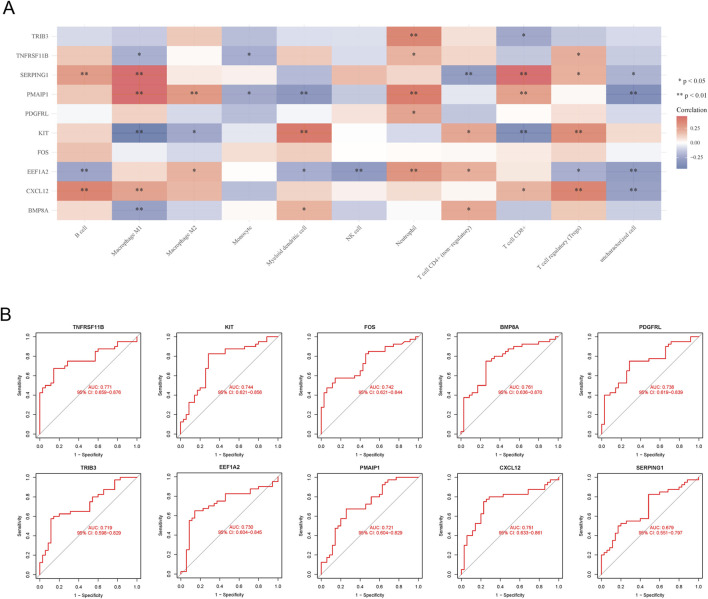
Immune cell correlation and ROC curves analysis of hub genes. **(A)** Immune cell correlation analysis of hub genes; **(B)** ROC curves analysis of hub genes.

Utilizing the MCP counter algorithm, immune infiltration and immune cell correlation analysis revealed the presence of 10 common immune cells. The analysis demonstrated that all 10 hub genes displayed diverse levels of correlation with these immune cells (Figure 4A). Additionally, the diagnostic efficacy of hub genes for FTC was evaluated using ROC curves, revealing that all hub genes had an AUC value >0.5 (Figure 4B).

### Expression verification of hub genes

**FIGURE 5:**
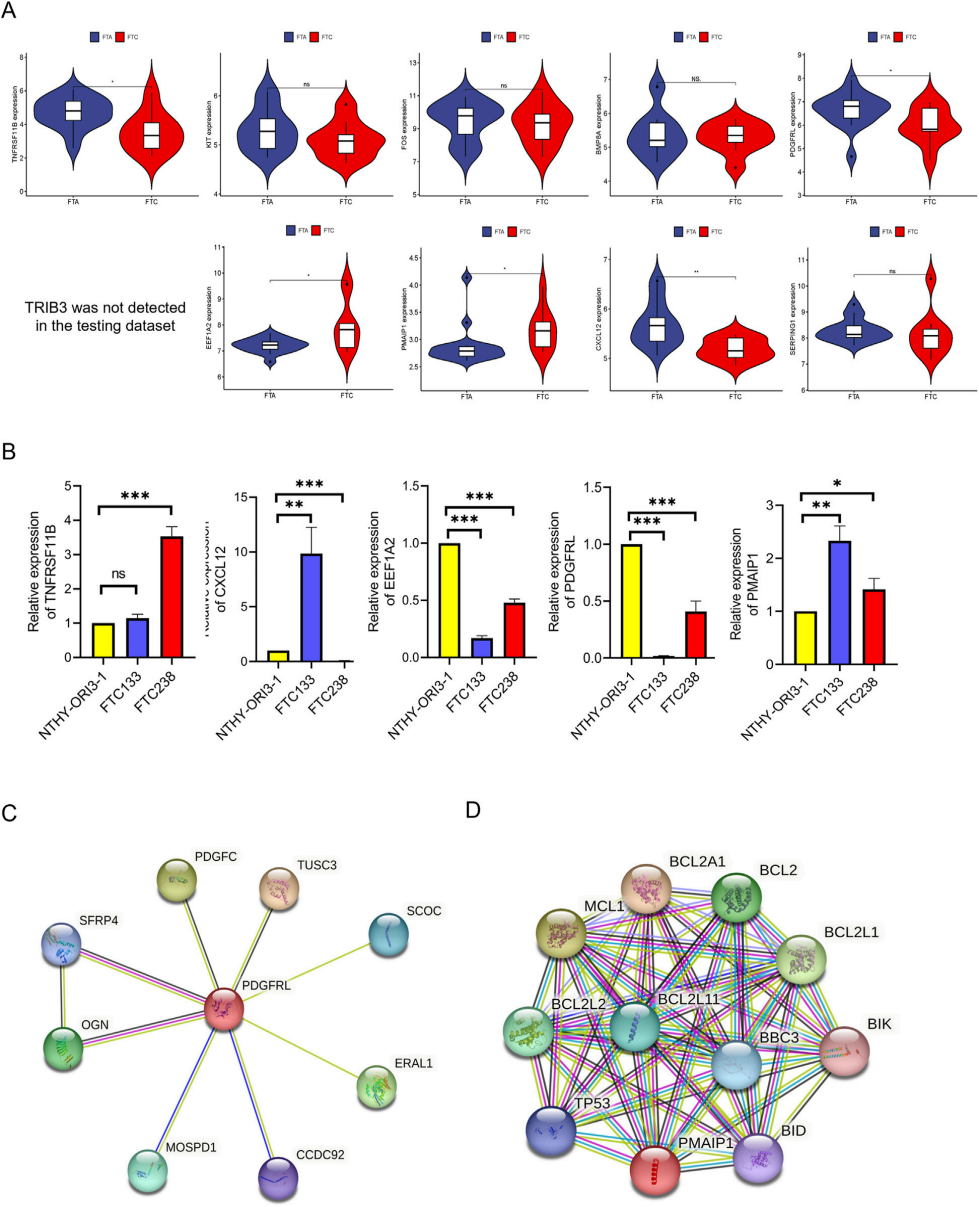
Identification of hub genes. **(A)** The expression level of hub genes between FTC and FTA in microarray data; **(B)** The mRNA levels of the hub genes were compared between FTC cell lines and NTHY-ORI3-1; **(C)** PPI analysis of PDGFRL; **(D)** PPI analysis of PMAIP1. (*P < 0.05, **P < 0.01, ***P < 0.001)

Further refinement of hub genes was conducted through two screening methods. First, we used GSE29315 as testing dataset, revealing that TRIB3 was not detected. Simultaneously, TNFRSF11B, CXCL12, EEF1A2, PDGFRL, and PMAIP1 exhibited differential expression (Figure 5A). Subsequently, qRT-PCR was utilized to assess the expression variances of these five candidate genes between FTC cell lines and NTHY-ORI3-1. The findings indicated that, among these five hub genes, only PMAIP1 and PDGFRL exhibited expression patterns consistent with the bioinformatics analysis results. Specifically, the expression level of PDGFRL decreased in two FTC cells compared to NTHY-ORI3-1, while the expression level of PMAIP1 increased in two FTC cells (Figure 5B). Furthermore, in order to investigate the potential functions of PMAIP1 and PDGFRL, we conducted separate PPI analyses for each (Figures 5C, D). Notably, only eight genes were found to potentially interact with PDGFRL.

### The expression level of PDGFRL and PMAIP1 in FTC and FTA tumor

**FIGURE 6:**
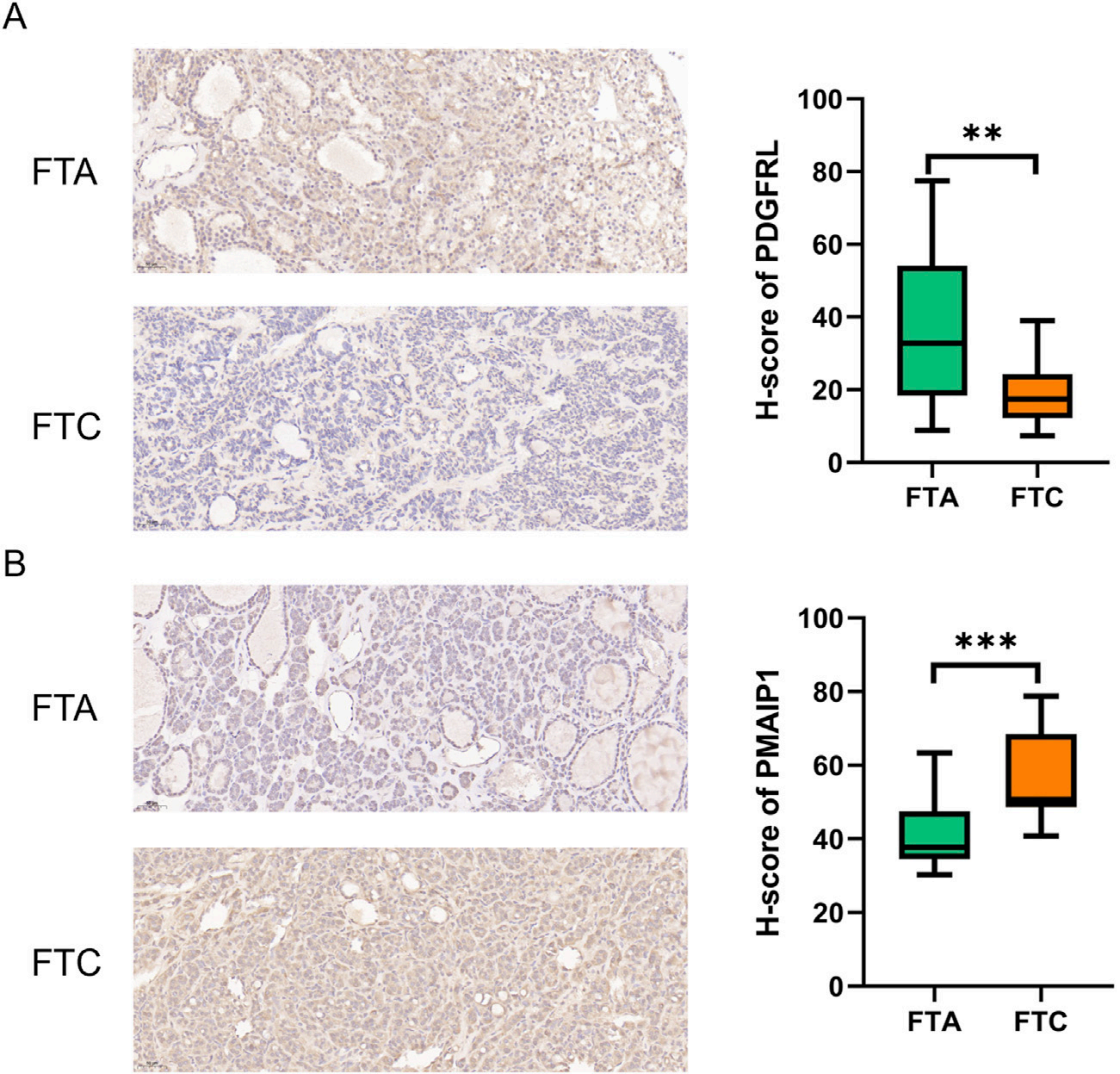
IHC was used to detect the difference expression of PMAIP1 and PDGFRL between FTC and FTA. **(A)** The protein level of PDGFRL in FTA and FTC; **(B)** The protein level of PMAIP1 in FTA and FTC. (**P < 0.01, ***P < 0.001)

We utilized six sections of FTC and six sections of FTA to assess the protein expression levels of PMAIP1 and PDGFRL by using IHC. Our findings suggest that, in comparison to FTA, the expression of PDGFRL is decreased in FTC, while PMAIP1 exhibits elevated expression levels in FTC (Figure 6). The results obtained from IHC were in agreement with those from bioinformatics analysis and qRT-PCR.

## Discussion

The diagnosis of FTC presented several challenges, particularly in distinguishing it from FTA, despite the ability to differentiate between the two based on classic histological criteria following surgery. The advancement of surgical techniques underscores the significance of accurately diagnosing FTC preoperatively to inform surgical decision-making and improve patient outcomes. Molecular markers have emerged as a promising avenue for enhancing diagnostic accuracy in clinical practice recently. This study utilized WES and microarray data to analyze the genome and transcriptome profiles of FTC and FTA. Immune-related gene sets were integrated into the analysis to identify a group of genes with potential diagnostic significance for FTC and FTA. Validation of these genes was conducted using a separate set of microarray data. Subsequent cellular and tissue level validation via qRT-PCR and IHC revealed abnormal expression of PMAIP1 and PDGFRL in FTC compared to FTA, suggesting their potential utility as immunodiagnostic markers. Additionally, our research encompassed a range of supplementary analyses targeting specific genes.

The differentiation between FTC and FTA is crucial for the accurate diagnosis and treatment of FTC, given its pathological characteristics. Unlike FTA, FTC is characterized by local invasion and distant metastasis. The exclusion of normal thyroid tissue in this study is justified by the distinct features of FTC/FTA compared to normal thyroid tissue. Therefore, achieving an accurate diagnosis of FTC is more paramount. In this study, we deviated from previous approaches by incorporating an immune gene set for screening purposes. Prior studies have indicated that analyzing various intratumoral and extratumoral regions can reveal specific tumor-immune characteristics at the invasion front, implying a potential link between tumor-immune profiles and the development and initial invasion of FTC (Saburi et al., 2022). Treatment with the PD-L1 inhibitor pembrolizumab demonstrated tolerable toxicity and sustained antitumor effects in a limited number of patients with advanced PTC and FTC (Oh et al., 2023). Consequently, the tumor-immune characteristics of FTC may be significant in its pathogenesis and offer a potential avenue for immunotherapy. AUC values for PMAIP1 and PDGFRL were determined to be 0.721 and 0.738, respectively, suggesting promising diagnostic potential for FTC. Additionally, given their classification as immune-related genes, it is plausible that they may contribute to the progression of FTC. Consequently, our study not only serves as a valuable resource for the accurate diagnosis of FTC, but also sets the groundwork for potential immunotherapeutic interventions targeting this disease. The inclusion of immune gene sets in our analysis was motivated by this rationale. Moving forward, further investigations will be conducted to elucidate the specific roles of PMAIP1 and PDGFRL, particularly in relation to the immune functionality of FTC. Furthermore, the detection of gene expression at the cellular level not only serves to validate their diagnostic significance but also plays a crucial role in guiding future molecular biology investigations.

A notable constraint observed in numerous studies comparing FTC and FTA lies in the limited sample sizes utilized. To address this issue, we conducted analyses on gene sets from the GEO database with the larger sample sizes available, specifically GSE82208, GSE27155, and GSE29315, which were utilized as both training and validation sets. Therefore, transcriptome sequencing data from 49 FTC and 52 FTA were utilized to validate differential expression of PMAIP1 and PDGFRL in order to reduce potential errors. The findings indicated an upregulation of PMAIP1 and a downregulation of PDGFRL in FTC compared to FTA. Additionally, ROC curve analysis confirmed the diagnostic utility of PMAIP1 and PDGFRL for distinguishing FTC. Despite the limited sample number of our study (6 FTC and 6 FTA), consistent results were obtained. Furthermore, PDGFRL was identified as a potential biomarker for endometriosis through analysis of GEO datasets, development of a diagnostic model, and RT-qPCR validation, also provide new insights into endometriosis treatment (Jiang et al., 2022). A separate study demonstrated a significant increase in the expression levels of PMAIP1 in invasive gonadotrophic pituitary adenomas compared to non-invasive adenomas (Yuan et al., 2023). This suggests that PMAIP1 and PDGFRL may serve as potential immune-biomarkers in various diseases, including FTC.

PPI analysis of PMAIP1 revealed close associations with BCL family proteins, as PMAIP1 was initially identified as an apoptosis-related gene with additional biological functions subsequently elucidated (Guikema, Amiot, and Eldering, 2017). Studies have confirmed the involvement of PMAIP1 in leukemia (Shah et al., 2023), pancreatic cancer (Doffo et al., 2022), pituitary adenoma (Asuzu et al., 2022) and triple-negative breast cancer (Cirillo et al., 2023), and other cancer types. Additionally, PMAIP1 has been identified as an immune gene with a confirmed role in immune function. NOXA (Aliases of PMAIP1) has been shown to impact anti-CART cell therapy, serving as a potential predictive marker for patient response and survival following CART cell transfusion. Targeting NOXA may enhance the therapeutic efficacy of CART cells (Yan et al., 2022). Collectively, these findings suggest that PMAIP1 may play a crucial role in apoptosis and immune response mechanisms, which also provides ideas for the development of new therapeutic modalities through targeting PMAIP1. Prior research has indicated that azacitidine has the ability to enhance the expression of PMAIP1, leading to heightened sensitivity of preclinical acute myeloid leukemia models to venetoclax. This suggests promising avenues for addressing resistance to current therapies for acute myeloid leukemia (Cojocari et al., 2021). Artemisinin, an antimalarial drug, has been found to induce NOXA to displace BIM in Mcl-1, leading to a synergistic induction of apoptosis with venetoclax. Research indicates that artemisinin acts as a connecting factor between venetoclax and cytarabine through NOXA and Bim-mediated apoptosis and Mcl-1 reduction. This mechanism reverses cytarabine-induced Mcl-1/p-Chk1 resistance by targeting the NOXA/Mcl-1/Bim axis, offering a novel triple combination therapy for acute myeloid leukemia (Zhang et al., 2022). Our study further revealed PMAIP1 is positively correlated with macrophage M1, macrophage M2, neutrophil, T cell CD8^+^ and negatively correlated with monocyte and myoid dendritic cells, indicating a potential role for PMAIP1 in modulating the disease process through immune cell regulation. Furthermore, the association of PMAIP1 with immune cell types suggests a potential immune regulatory function. Based on our results, we boldly hypothesize that PMAIP1 plays an important regulatory role in the homeostasis of the immune microenvironment of FTC, and this function may be mainly accomplished through macrophages. It is worth noting that the analysis results show that there is no significant correlation between PMAIP1 and CD4^+^ T cells, because the number of CD8^+^ T cells, which belong to the same category of immune cells, increases with the upregulation of PMAIP1 expression. This is very interesting. In addition, changes in the expression level of PMAIP1 will affect the quantity and proportion of various immune cells in the patient’s tumor tissue. This indicates that targeting PMAIP1 may achieve the treatment of FTC by altering the tumor immune microenvironment.

The downregulation of PDGFRL expression in FTC aligns with findings from prior research, suggesting its role as a tumor suppressor gene. Overexpression of PDGFRL has been shown to impede the proliferation of HCS-2/8 in human chondrosarcoma cells (Kawata et al., 2017). However, there is limited literature on the molecular function and mechanism of PDGFRL. Our analysis of PDGFRL using PPI data revealed only eight potentially related genes, supporting this hypothesis.

The findings derived from analyses utilizing data from the GEO and TCGA databases indicate that PDGFRL exhibits strong predictive capabilities for overall survival in gastric cancer across five independent cohorts. Furthermore, the risk score demonstrates a clear positive association with macrophage abundance (Huo et al., 2021). This finding contrasts with our initial analysis results. Our results suggested a significant correlation between PDGFRL expression levels and infiltration of neutrophil, while no such correlation is observed with macrophages. It is essential to acknowledge that while findings may vary across studies, the fundamental relationship between PDGFRL and the immune system remains unequivocal. Unlike PMAIP1, in FTC, only a positive correlation between PDGFRL and neutrophils was observed, and there was no significant association with other immune cells. This is a question worthy of in-depth consideration. Does it mean that in the immune microenvironment of FTC, the role of PDGFRL is limited, that is, it can only be regulated through neutrophils? Considering that the PPI analysis of PDGFRL only yielded relatively few results, our hypothesis is plausible. This issue requires further exploration and verification. Notably, A genome-wide epistasis study of COVID-19 severity was conducted on a cohort of 2,243 patients with severe symptoms and 12,612 patients with no or mild symptoms from the United Kingdom Biobank, followed by a replication study in an independent Spanish cohort consisting of 1,416 cases and 4,382 controls (Lin et al., 2023). The study revealed a significant interaction between rs9792388 upstream of PDGFRL and rs3025892 downstream of SNAP25, suggesting a potential association with an increased risk of severe disease. This finding highlighted the potential utility and relevance of PDGFRL in various fields.

Notably, our research has uncovered an intriguing phenomenon of contradictory expression patterns of PMAIP1 and PDGFRL in FTC238 vs. FTC133. While our initial objective was to analyze the expression variances of PDGFRL and PMAIP1 in FTC cell lines compared to normal thyroid cell lines, our study did not specifically investigate the differences in expression levels between FTC133 and FTC238. Nevertheless, we observed a notable discrepancy in the expression levels of PDGFRL and PMAIP1 between these 2 cell lines. Specifically, compared with FTC133, PDGFRL exhibited higher expression in FTC238, while PMAIP1 showed lower expression in FTC238. Given that FTC238 originates from lung metastasis and is presumed to be more aggressive than FTC133. We are intrigued by this finding and hypothesize that it may be attributed to the distinct characteristics of FTC133 and FTC238. This issue deserves further exploration in the future.

In our present study, there were several limitations. Firstly, the findings of this study, conducted with a restricted participant pool, necessitate additional confirmation in a larger cohort of individuals diagnosed with FTC and FTA. Secondly, the scarcity of FTC cases hindered the procurement of an adequate number of tissue samples for transcriptome sequencing, prompting the utilization of data from the GEO. Lastly, the focus of the study was on identifying diagnostic markers for FTC and FTA. However, the absence of FTA cell lines limited the assessment to the expression levels of hub genes in FTC cell lines compared to a normal thyroid cell line. These matters will be examined in forthcoming research endeavors, as we aim to gather more samples in order to corroborate our findings and investigate the roles and mechanisms of PMAIP1 and PDGFRL in FTC.

PMAIP1 and PDGFRL have been validated as potential diagnostic markers for FTC, holding significant importance in guiding its clinical diagnosis. In clinical practice, we anticipate that the findings of our study will enhance preoperative diagnosis of FTC, inform the scope of surgical intervention, and improve patient prognosis. Moreover, we plan to explore additional possibilities. For instance, we aim to detect the expression levels of PMAIP1 and PDGFRL in patients’ blood or hair samples. Currently, the underlying mechanisms of these two biomarkers remain unclear. In our subsequent research, we will primarily focus on the roles and specific mechanisms of PMAIP1 and PDGFRL in FTC. We’ll also examine their relationships with clinicopathological features and patient prognosis. Our goal is to uncover more potential applications beyond diagnostic capabilities, such as targeted therapies.

In summary, our investigation has indicated that PMAIP1 and PDGFRL exhibit potential as immunodiagnostic indicators for distinguishing between FTC and FTA with a high degree of precision. As we known, it is the first research to identify a potential relationship between PMAIP1 and PDGFRL in relation to FTC. This conclusion, drawn from an analysis of the three largest microarray datasets of FTC and FTA, was further confirmed through the examination of an independent collection of FFPE samples and 3 cell lines. The potential utility of this molecular test as a valuable adjunct for pathologists in cases of ambiguous thyroid follicular neoplasms, where histopathological criteria alone may not provide a definitive diagnosis, warrants further investigation.

## Data Availability

The data presented in the study are deposited in the NCBI repository, accession number PRJNA1261386.
